# Real-world outcomes of Adjuvant De Gramont versus Xelox chemotherapy in reSected gasTric cancER: a propensity score-matched analysis (ASTER study)

**DOI:** 10.1038/s41417-025-00945-1

**Published:** 2025-07-26

**Authors:** Ina Valeria Zurlo, Fausto Rosa, Diana Giannarelli, Giovanni Trovato, Massimiliano Salati, Andrea Spallanzani, Michele Basso, Carmelo Pozzo, Sergio Alfieri, Giampaolo Tortora, Antonia Strippoli

**Affiliations:** 1 Medical Oncology Unit, Comprehensive Cancer Center, Fondazione Policlinico Universitario “A. Gemelli” IRCCS, Rome, Italy; 2 Medical Oncology Unit, “Vito Fazzi” Hospital, Lecce, Italy; 3 Department of Surgery, Fondazione Policlinico Universitario “A. Gemelli” IRCCS, Rome, Italy; 4 Università Cattolica del Sacro Cuore, Rome, Italy; 5 Fondazione Policlinico Universitario Agostino Gemelli IRCCS—Gemelli Generator—Facility of Epidemiology and Biostatistics, Rome, Italy; 6 Division of Oncology, Department of Oncology and Hematology, University Hospital of Modena, Modena, Italy

**Keywords:** Gastrointestinal cancer, Drug development

## Abstract

The role of adjuvant chemotherapy (aCT) in gastric and esophago-gastric junction cancer (GC/EGJC) remains controversial. This study (ASTER study) aimed to compare the clinical outcomes of De Gramont (DG) versus XELOX/FOLFOX (OXA) regimens in a European real-world setting. This retrospective, bicentric study included patients treated with aCT between January 2001 and January 2018. A propensity score-matched (PSM) analysis was performed to compare oncological outcomes between DG and OXA regimens. Primary endpoints were disease-free survival (DFS) and overall survival (OS). Statistical analyses included the chi-square test, Kaplan–Meier method, and Cox proportional hazards modeling. Among 255 patients (127 DG, 128 OXA), 160 were matched (80 per arm) by PSM. Median DFS and OS did not differ significantly between groups (mDFS: 102.3 vs. 85.4 months, *p* = 0.91; mOS: 119.5 vs. 89.8 months, *p* = 0.69). In PSM-adjusted analysis, DG showed a trend towards longer DFS (*p* = 0.052) and significantly improved OS (*p* = 0.016). Multivariate analysis confirmed age, ECOG PS, resection margins, and stage as major prognostic factors. DG and OXA regimens demonstrated similar efficacy in the adjuvant treatment of resected GC/GEJC in a European cohort. Further prospective studies are warranted to optimize regimen selection and refine patient stratification.

## Introduction

Gastric and esophago-gastric junction cancer (GC/EGJC) represent major global health challenges, ranking fifth in incidence and fourth in mortality worldwide [1].

Despite surgical resection remaining the cornerstone and only potentially curative treatment for GC [2], disease relapse occurs in 40–80% of patients [3]. Adjuvant chemotherapy (aCT) has been shown to improve survival compared to surgery alone, particularly in Asian populations, as evidenced by several meta-analyses and randomized trials [4–7]. Adjuvant chemotherapy (aCT) has been shown to improve survival compared to surgery alone, particularly in Asian populations, as evidenced by several meta-analyses and randomized trials [4–7]. A comprehensive meta-analysis including 17 randomized controlled trials confirmed that aCT significantly benefits overall survival (OS) and disease-free survival (DFS) [6].

Nevertheless, the optimal adjuvant regimen remains undefined. Current national and international guidelines differ in their recommendations. The European Society for Medical Oncology (ESMO) advocates fluoropyrimidine-based chemotherapy for patients with ≥Stage IB GC who undergo upfront surgery without preoperative chemotherapy, while recommending S-1 following D2 resection for Asian patients [8]. Conversely the National Comprehensive Cancer Network (NCCN) supports capecitabine plus oxaliplatin following primary D2 lymphadenectomy [9]. Recent network meta-analyses have addressed the relative efficacy of aCT, adjuvant radiotherapy (aRT) and adjuvant radiochemotherapy (aRTCT) in resected GC, but failed to adequately compare different aCT regimens [10, 11]. Moreover, updated results from randomized controlled trials (RCTs) and emerging studies continue to shape the landscape [12, 13], although uncertainties persist regarding treatment rankings and effect estimates [14, 15].

The MAGIC trial in 2006 revolutionized GC management by introducing perioperative chemotherapy as a strategy to improve survival through tumor downstaging, micrometastasis control, and assessment of chemosensitivity [16–18]. Consequently, perioperative chemotherapy combined with D2 lymphadenectomy became standard practice for locally advanced GC in Europe [19]. The FLOT4 trial later established a new triplet regimen—docetaxel, oxaliplatin, and 5-fluorouracil—as a superior perioperative option, replacing the ECF/ECX regimens [20].In contrast, Asian countries, upfront radical gastrectomy with D2 dissection remains standard, with adjuvant S-1 or XELOX regimens demonstrating survival benefits [21–23].

Prior to the widespread adoption of perioperative strategies, postoperative chemotherapy—often monotherapy with fluoropyrimidines or doublet XELOX regimens—was employed in Europe, adapting schedules validated primarily in Asian populations. However, direct data in Western cohorts are limited.

This study retrospectively examined the outcomes of resected GC/EGJC patients treated with adjuvant chemotherapy at two Italian centers. Specifically, it compared the De Gramont (DG) regimen and the XELOX/FOLFOX regimen (OXA) using a propensity score-matching (PSM) analysis, aiming to provide real-world evidence regarding the efficacy of these two commonly adopted strategies in a European setting.

## Materials and methods

This observational, retrospective, bicentric study (ASTER study) included patients with histologically confirmed GC or EGJC treated at two Italian oncology centers (Fondazione Policlinico Universitario “A. Gemelli”–IRCCS and Policlinico Universitario of Modena) between January 2001 and January 2018. Eligible patients had undergone either total or subtotal gastrectomy and subsequently received aCT with either DG regimen or an oxaliplatin-based doublet regimen (OXA arm).

Inclusion criteria were as follows:

- Histologically confirmed resected GC or EGJC.
- Total or Subtotal gastrectomy performed with curative intent.
- Age ≥18 years at the time of aCT initiation.
- Administration of aCT (DG or OXA regimen).
- Available histopathological classification.
- Eastern Cooperative Oncology Group (ECOG) performance status (PS) of 0–2.
- Adequate bone marrow, liver, and renal function.
- Absence of serious comorbidities affecting treatment feasibility or short-time survival.
- Signed informed consent for CT and retrospective clinical data analysis.
  Considering the period of enrollment, we did not collect data on microsatellite instability (MSI), as it was not a mandatory requirement at that time.
  Exclusion criteria included:
- History of metastatic disease or another malignancy diagnosed within the previous 5 years (excluding non-melanoma skin cancer or in situ cervical cancer).
- Administration of perioperative chemotherapy, or upfront surgery without subsequent adjuvant therapy.

Pathological staging was assigned according to standard TNM criteria, and distant metastases were ruled out using contrast-enhanced computer tomography (ce-CT) scan.

Grade 3 and 4 adverse events (AEs) were systematically prospectively collected and reported in accordance with the Common Terminology Criteria for Adverse Events (CTCAE) v4.0.

All patients data were collected anonymously. The study was conducted in accordance with the rules of the local Ethics Committee and the Declaration of Helsinki and was approved by the local Ethics Committee of Fondazione Policlinico “A. Gemelli” IRCCS (Prot.- ID 6723).

### Treatment procedures

All patients underwent total or subtotal gastrectomy with lymphadenectomy prior to initiating aCT. Treatment regimens included either a fluoropyrimidine monotherapy protocol (DG regimen) or an oxaliplatin-based doublet regimen (OXA arm). The OXA arm included either the FOLFOX-6 schedule (leucovorin plus oxaliplatin and bolus/infusional 5-fluorouracil), or the XELOX schedule (capecitabine plus oxaliplatin).

Adjuvant chemotherapy was administered for a maximum duration of 6 months, corresponding to 12 cycles for the DG or FOLFOX-6 regimens and 8 cycles for the XELOX regimen.

Surgical margins were classified as follows:

- R0 resection: no microscopic tumor involvement of proximal, distal, or circumferential margins.
- R1 resection: presence of microscopic residual tumor at any surgical margin.

Post-treatment follow-up adhered to international guidelines and included clinical evaluation and imaging (CT-scans or abdominal ultrasound) every 6 months.

### Statistical analysis

To minimize the effects of potential confounding factors and enhance the accuracy of treatment effect estimation, a PSM approach was applied to balance baseline characteristics between the DG and OXA treatment groups.

The primary objectives of the study were to evaluate disease-free survival (DFS) and overall survival (OS) according to adjuvant chemotherapy regimen. Tolerability was also assessed. The variables considered for PSM included: gender, age, sex, ECOG PS, histological subtype, presence of signet ring cells, tumor grading, lymphovascular invasion (LVI), resection margin status, tumor location, type of surgery, extent of lymphadenectomy, pathological stage, T stage, N stage, administration of aRT.

Baseline covariate imbalances between the two groups were assessed using chi-square tests for individual variables and multivariable logistic regression analysis for overall covariate balance. A *p* value < 0.10 was considered indicative of imbalance.

The PSM—the probability of receiving either the DG or OXA regimen based on baseline characteristics—was calculated using a multivariable logistic regression model [24].

A 1:1 nearest-neighbor matching without replacement was performed, using a caliper width of 0.8 standard deviations of the logit of the PSM [25].

Subgroup analyses were conducted exclusively within the propensity score-matched cohort, given the limited statistical power of the unmatched subgroup analyses.

Graphical methods were used to assess the distribution of propensity scores before and after matching.

For survival analysis, the Kaplan–Meier method was used to estimate DFS and OS, and differences between groups were evaluated using the log-rank test. Stratified Cox proportional hazards regression models, accounting for matched pairs, were employed to estimate hazard ratios (HRs) and 95% confidence intervals (CIs).

DFS was defined as the time from surgery to either disease recurrence (local or distant), death from any cause, or last follow-up. OS was defined as the time from primary diagnosis to death from any cause. Patients without events were censored at the time of last known follow-up. All statistical analyses were performed using R software version 4.1.2 (library MatchIt) and IBM SPSS Statistics for Windows, v.28.0 (Armonk, NY).

## Results

### Patients characteristics

A total of 255 consecutive patients with stage IB, II, or III GC or EGJC treated at the Medical Oncology Units of Fondazione Policlinico Universitario “A. Gemelli” IRCCS and University Hospital of Modena between January 2001 and January 2018 were included in the analysis.

The median patient age was 66 years (range: 27–82 years). Twenty-eight patients had an ECOG PS of 2. Histologically, 129 patients presented with a diffuse type, 126 with an intestinal type, and 59 exhibited signet-ring cell features (including 2 within the intestinal-type group). High-grade (G3) histology was reported in 181 patients, and 84 patients were positive for LVI.

Tumor location involved the gastro-esophageal junction or cardia in 33 patients. A total of 96 patients underwent total gastrectomy, while 23 patients received a D1 lymphadenectomy. Stage III disease was present in 152 patients, with 203 patients exhibiting T3–T4 primary tumors, and 150 patients demonstrating N2–N3 nodal involvement. Additionally, 76 patients received aRT.

Regarding chemotherapy regimens, 127 patients received the De Gramont (DG) regimen, while 128 patients were treated with an oxaliplatin-based doublet regimen (OXA arm).

Detailed baseline characteristics of the overall cohort and according to treatment arm are presented in Table 1.

**Table 1:** Patients’ characteristics in DG and OXA arm and unbalanced variables in the two arms: ECOG PS, LVI, resection margin, lymphadenectomy, stage, T and N involvement.

	DG arm (%)	OXA arm (%)	*p* value
Gender			
Male	70 (48.6)	74 (51.4)	0.66
Female	57 (51.4)	54 (48.6)	
Age (years)			
<66	71 (50.0)	71 (50.0)	0.94
≥66	56 (49.6)	57 (50.4)	
ECOG PS			
0	70 (37.2)	118 (62.8)	<0.0001
1–2	57 (85.1)	10 (14.9)	
Lauren			
Intestinal	63 (50.0)	63 (50.0)	0.95
Diffuse	64 (49.6)	65 (50.4)	
Signet ring cells			
No	95 (48.5)	101 (51.5)	0.44
Yes	32 (54.2)	27 (45.8)	
Grading			
G1–G2	40 (54.1)	34 (45.9)	0.38
G3	87 (48.1)	94 (51.9)	
LVI			
No	98 (57.3)	73 (42.7)	0.001
Yes	29 (34.5)	55 (65.5)	
Resection margin			
R0	123 (51.5)	116 (48.5)	0.04
R1	4 (25.0)	12 (75.0)	
Tumor location			
Upper	16 (48.5)	17 (51.5)	0.87
Medium/Lower	111 (50.0)	111 (50.0)	
Type of surgery			
Subtotal-gastrectomy	81 (50.9)	78 (49.1)	0.64
Total-gastrectomy	46 (47.9)	50 (52.1)	
Lymphadenectomy			
D2	106 (45.7)	126 (54.3)	<0.0001
D1	21 (91.3)	2 (8.7)	
Stage			
I–II	72 (69.9)	31 (30.1)	<0.0001
III	55 (36.2)	97 (63.8)	
T involvement			
T1–T2	39 (75.0)	13 (25.0)	<0.0001
T3–T4	88 (43.3)	115 (56.7)	
N involvement			
N0–N1	64 (61.0)	41 (39.0)	0.003
N2–N3	63 (42.0)	87 (58.0)	
aRT			
No	85 (47.5)	94 (52.5)	0.26
Yes	42 (55.3)	34 (44.7)	
Grade 3–4 AEs			
No	115 (90.5)	107 (83.6)	0.09
Yes	12 (9.5)	21 (16.4)	

*DG* De Gramont, *OXA* XELOX/FOLFOX, *LVI* lymphovascular invasion, *ECOG PS* Eastern Cooperative Oncology Group (ECOG) performance status, *aRT* adjuvant radiotherapy, *AEs* adverse events.

### Outcomes

**Fig. 1:**
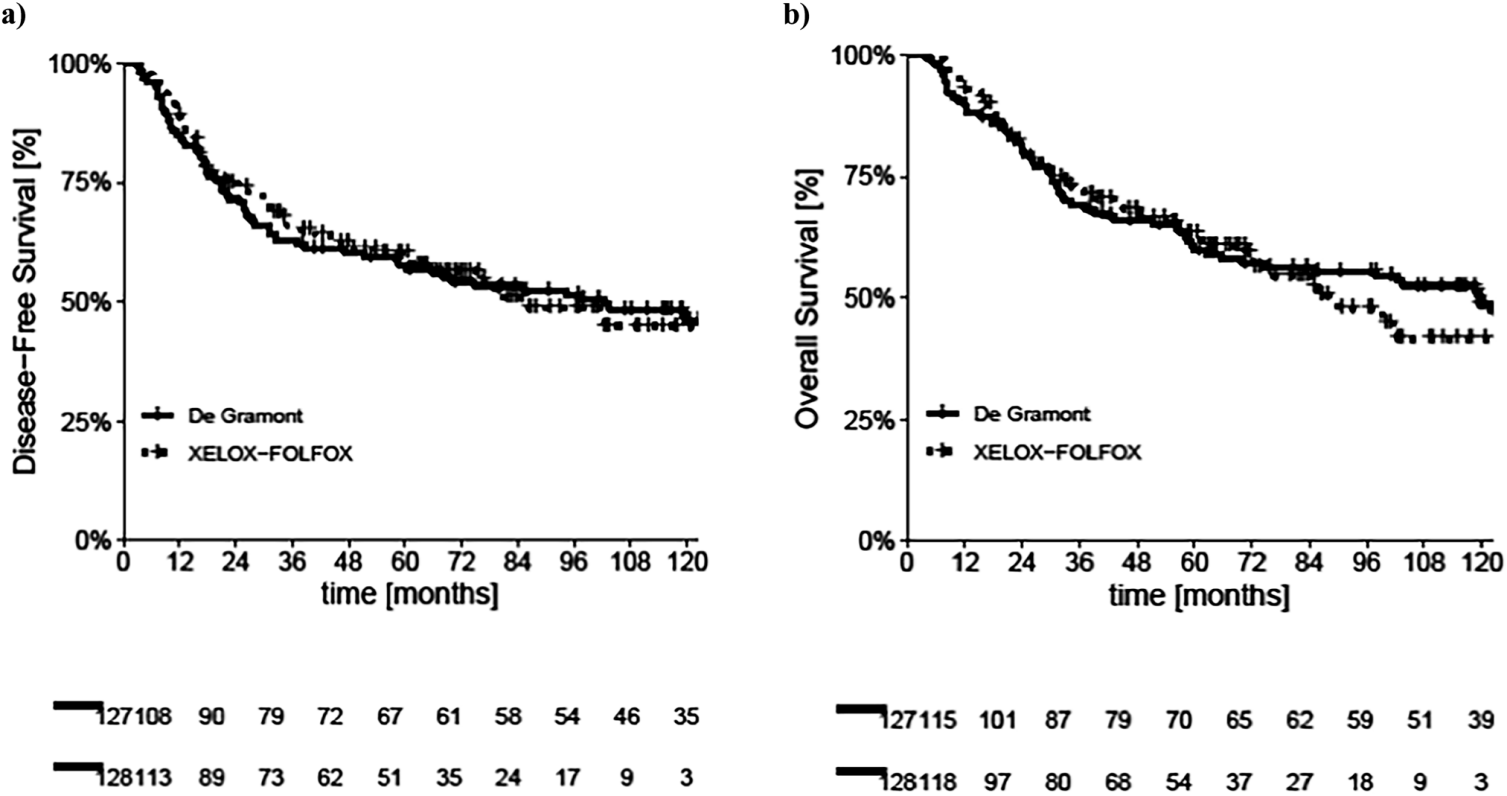
Kaplan-Meier curves for DFS and OS before PSM. **a** Kaplan–Meier curves for DFS before PSM in DG and OXA arm. **b** Kaplan–Meier curves for OS before PSM in DG and OXA arm.

In the overall study population, the median DFS (mDFS) was 102.3 months (95% CI: 48.4–156.2) in DG arm and 85.4 months (95% CI: 57.9–112.9) in the OXA arm, with no statistically significant difference observed between the two groups (*p* = 0.91) as shown in Fig. 1a.

Similarly, the median OS (mOS) was 119.5 months (95% CI: 72.2–166.8) in DG arm and 89.8 months (95% CI: 62.1–117.5) in the OXA arm, without a significant differences (*p* = 0.69) as shown in Fig. 1b.

Univariate Cox regression analysis identified several variables associated with DFS, including gender (*p* = 0.01), age (*p* < 0.0001), ECOG PS (*p* = 0.018), tumor grading (*p* < 0.0001), LVI (*p* = 0.001), resection margin status (*p* < 0.0001), tumor location (*p* < 0.0001), type of surgery (*p* = 0.002), pathological stage (*p* < 0.0001), T stage (*p* < 0.0001), N stage (*p* < 0.0001), and receipt of aRT (*p* = 0.017).

At multivariate analysis, DFS remained significantly associated with age (*p* = 0.001), ECOG PS (*p* = 0.037), tumor grading (*p* = 0.01), resection margin status (*p* < 0.0001), tumor location (*p* = 0.001), pathological stage (*p* < 0.0001), and chemotherapy regimen (*p* = 0.035) (Table 2).

**Table 2:** Variables influencing disease-free survival (before PSM).

	Univariate	Multivariable with all variables	Multivariable with selection
Gender (male vs. female)	1.62 (1.12–2.35) *p* = 0.01	1.41 (0.94–2.11) *p* = 0.10	
Age (≥66 vs. <66)	1.91 (1.33–2.73) *p* < 0.0001	1.78 (1.21–2.64) *p* = 0.004	1.91 (1.32–2.76) *p* = 0.001
ECOG PS (1–2 vs. 0)	1.58 (1.08–2.29) *p* = 0.018	1.59 (1.00–2.53) *p* = 0.052	1.59 (1.03–2.47) *p* = 0.037
Lauren (diffuse vs. intestinal)	1.01 (0.71–1.44) *p* = 0.95	0.84 (0.54–1.32) *p* = 0.45	
Signet ring cells (yes vs. no)	1.20 (0.81–1.78) *p* = 0.37	1.64 (1.01–2.68) *p* = 0.046	
Grading (G3 vs. G1–G2)	2.36 (1.49–3.74) *p* < 0.0001	1.51 (0.90–2.54) *p* = 0.12	1.85 (1.15–2.98) *p* = 0.01
LVI (yes vs. no)	1.84 (1.28–2.64) *p* = 0.001	1.39 (0.91–2.11) *p* = 0.13	
Resection Margin (R1 vs. R0)	3.93 (2.23–6.93) *p* < 0.0001	3.68 (1.87–7.25) *p* < 0.0001	4.24 (2.31–7.80) *p* < 0.0001
Tumor location (medium/lower vs. upper)	0.43 (0.28–0.67) *p* < 0.0001	0.53 (0.32–0.87) *p* = 0.012	0.48 (0.31–0.76) *p* = 0.001
Type of surgery (TG vs. STG)	1.74 (1.22–2.48) *p* = 0.002	1.24 (0.82–1.39) *p* = 0.30	
Lymphadenectomy (D1 vs. D2)	0.98 (0.55–1.75) *p* = 0.95	1.10 (0.55–2.18) *p* = 0.78	
Stage (III vs. I–II)	3.08 (2.04–4.64) *p* < 0.0001	3.22 (1.59–6.52) *p* = 0.001	3.22 (2.09–4.97) *p* < 0.0001
T involvement (T3–T4 vs. T1–T2)	3.06 (1.72–5.45) *p* < 0.0001	1.84 (0.96–3.52) *p* = 0.065	
N involvement (N2–N3 vs. N0–N1)	2.14 (1.46–3.14) *p* < 0.0001	0.87 (0.47–1.62) *p* = 0.66	
aCT regimen (OXA vs. DG)	0.98 (0.68–1.41) *p* = 0.91	0.58 (0.37–0.92) *p* = 0.021	0.62 (0.40–0.97) *p* = 0.035
aRT (yes vs. no)	1.55 (1.08–2.21) *p* = 0.017	1.05 (0.69–1.60) *p* = 0.81	
Grade 3–4 AEs (yes vs. no)	1.45 (1.09–2.06) *p* = 0.07	1.14 (0.77–1.68) *p* = 0.52	

*DG* De Gramont, *OXA* XELOX/FOLFOX, *ECOG PS* Eastern Cooperative Oncology Group (ECOG) performance status, *TG* total gastrectomy, *STG* sub-total gastrectomy, *LVI* lymphovascular invasion, *aCT* adjuvant chemotherapy, *aRT* adjuvant radiotherapy, *AEs* adverse events.

Regarding OS, univariate analysis showed significant associations with gender (*p* = 0.026), age (*p* < 0.0001), ECOG PS (*p* = 0.032), tumor grading (*p* < 0.0001), LVI (*p* < 0.0001), resection margin status (*p* < 0.0001), tumor location (*p* < 0.0001), type of surgery (*p* = 0.002), pathological stage (*p* < 0.0001), T stage (*p* < 0.0001), N stage (*p* < 0.0001), and receipt of aRT (*p* = 0.004).

At multivariate analysis, OS was significantly influenced by age (*p* < 0.0001), ECOG PS (*p* = 0.001), presence of signet-ring cells (*p* = 0.03), tumor grading (*p* = 0.03), resection margin status (*p* < 0.0001), tumor location (*p* = 0.001), pathological stage (*p* < 0.0001), and T stage (*p* = 0.025) (Table 3).

**Table 3:** Variables influencing overall survival (before PSM).

	Univariate	Multivariable with all variables	Multivariable with selection
Gender (male vs. female)	1.53 (1.05–2.21) *p* = 0.026	1.38 (0.92–2.08) *p* = 0.12	
Age (≥66 vs. <66)	2.14 (1.48–3.09) *p* < 0.0001	2.07 (1.41–3.05) *p* < 0.0001	2.24 (1.53–3.29) *p* < 0.0001
ECOG PS (1–2 vs. 0)	1.52 (1.04–2.22) *p* = 0.032	1.49 (0.93–2.39) *p* = 0.10	1.93 (1.29–2.87) *p* = 0.001
Lauren (diffuse vs. intestinal)	0.99 (0.69–1.42) *p* = 0.95	0.78 (0.50–1.22) *p* = 0.28	
Signet ring cells (yes vs. no)	1.22 (0.81–1.82) *p* = 0.34	2.04 (1.23–3.36) *p* = 0.006	1.60 (1.05–2.46) *p* = 0.03
Grading (G3 vs. G1–G2)	2.43 (1.51–3.89) *p* < 0.0001	1.69 (0.99–2.87) *p* = 0.053	1.71 (1.05–2.77) *p* = 0.03
LVI (yes vs. no)	1.94 (1.34–2.80) *p* < 0.0001	1.24 (0.82–1.88) *p* = 0.31	
Resection Margin (R1 vs. R0)	4.13 (2.34–7.31) *p* < 0.0001	3.28 (1.71–6.28) *p* < 0.0001	3.44 (1.89–6.29) *p* < 0.0001
Tumor location (medium/lower vs. upper)	0.43 (0.28–0.68) *p* < 0.0001	0.53 (0.31–0.88) *p* = 0.014	0.44 (0.28–0.70) *p* = 0.001
Surgery (TG vs. STG)	1.75 (1.22–2.51) *p* = 0.002	1.21 (0.79–1.84) *p* = 0.38	
Lymphadenectomy (D1 vs. D2)	0.82 (0.44–1.52) *p* = 0.52	0.94 (0.45–1.96) *p* = 0.86	
Stage (III vs. I–II)	3.30 (2.16–5.05) *p* < 0.0001	3.19 (1.58–6.42) *p* = 0.001	2.71 (1.72–4.26) *p* < 0.0001
T involvement (T3–T4 vs. T1–T2)	3.21 (1.76–5.84) *p* < 0.0001	1.95 (1.00–3.82) *p* = 0.051	2.11 (1.10–4.03) *p* = 0.025
N involvement (N2–N3 vs. N0–N1)	2.27 (1.53–3.36) *p* < 0.0001	0.92 (0.49–1.71) *p* = 0.79	
aCT regimen (OXA vs. DG)	1.08 (0.74–1.57) *p* = 0.69	0.62 (0.38–0.99) *p* = 0.046	
aRT (yes vs. no)	1.69 (1.18–2.43) *p* = 0.004	1.25 (0.82–1.89) *p* = 0.30	
Grade 3–4 AEs (yes vs. no)	1.40 (0.98–2.01) *p* = 0.06	1.20 (0.82–1.78) *p* = 0.35	

*ECOG PS* Eastern Cooperative Oncology Group (ECOG) performance status, *TG* total gastrectomy, *STG* sub-total gastrectomy, *LVI* lymphovascular invasion, *DG* De Gramont, *OXA* XELOX/FOLFOX, *aCT* adjuvant chemotherapy, *aRT* adjuvant radiotherapy, *AEs* adverse events.

### Propensity score matching analysis

A significant imbalance was observed between the DG and OXA groups in baseline characteristics prior to matching, specifically regarding ECOG performance status (*p* < 0.0001), presence of LVI (*p* = 0.001), resection margin status (*p* = 0.04), extent of lymphadenectomy (*p* < 0.0001), pathological stage (*p* < 0.0001), T stage (*p* < 0.0001), and N stage (*p* = 0.003) (Table 1).

Multivariate analysis further confirmed imbalances in ECOG performance status, lymphadenectomy, stage, and LVI distribution between the treatment arms.

Using these variables, a PSM sensitivity analysis was performed resulting in 80 patients successfully matched in each treatment group (DG and OXA arms).

The median follow-up in this subgroup was 68 months.

**Fig. 2:**
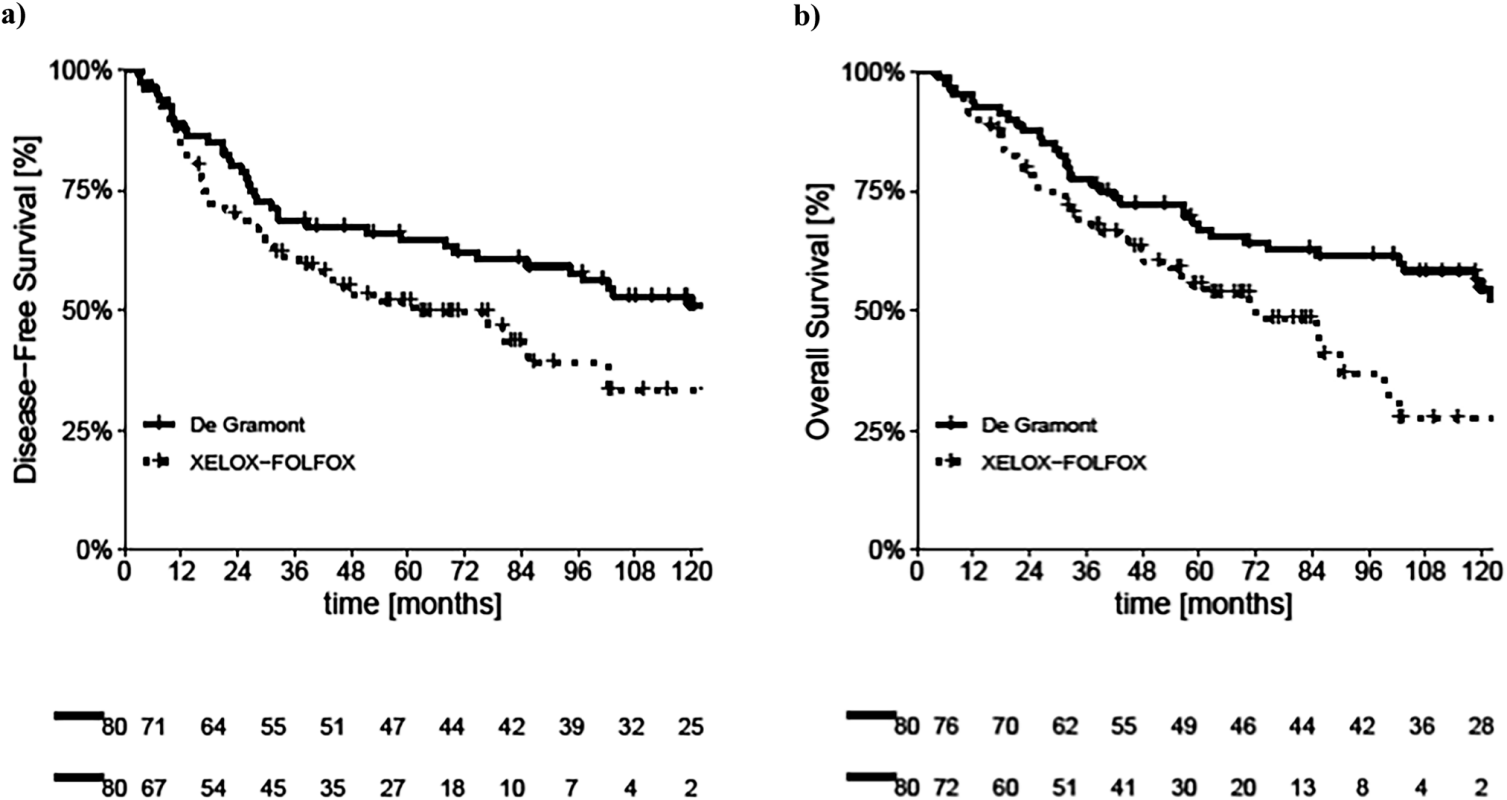
Kaplan-Meier curves for DFS and OS after PSM. **a** Kaplan–Meier curves for DFS after PSM in DG and OXA arm. **b** Kaplan–Meier curves for OS after PSM in DG and OXA arm.

In the PSM-adjusted cohort, a trend toward longer DFS was observed in DG arm compared to the OXA arm, though the difference was only marginally statistically significant (mDFS 133.2 vs. 61.4 months; hazard ratio [HR] 1.56; 95% CI 0.99–2.46; *p* = 0.052) as shown in Fig. 2a.

For OS, a statistically significant difference favoring the DG regimen was observed after matching (mOS 133.2 vs. 72.0 months respectively; hazard ratio [HR] 1.78; 95% CI 1.11–2.84; *p* = 0.016) as shown in Fig. 2b.

### Multivariate analysis

After propensity score-matching, multivariable Cox regression analysis was per-formed to adjust for residual imbalances between the treatment groups.

For DFS, several factors remained independently associated with outcomes, including:

- Gender (HR 1.51, 95% CI: 1.01–2.24; *p* = 0.043),
- Age (HR 1.90, 95% CI: 1.30–2.77; *p* = 0.001),
- ECOG performance status (HR 2.22, 95% CI: 1.48–3.34; *p* < 0.0001),
- Presence of LVI (HR 1.61, 95% CI: 1.10–2.36; *p* = 0.014),
- Resection margin status (HR 4.14, 95% CI: 2.27–7.54; *p* < 0.0001),
- Tumor location (HR 0.50, 95% CI: 0.31–0.78; *p* = 0.003),
- Pathological stage (Table 4).

**Table 4:** Univariate and multivariate analysis for DFS with selection of parameters statistically significant at the logistic regression after PSM.

	Univariate	Multivariable with selection
Gender (m vs. f)	2.19 (1.37–3.49) *p* = 0.001	2.21 (1.37–3.57) *p* = 0.001
Age (≥66 vs. <66)	1.92 (1.23–2.98) *p* = 0.004	1.69 (1.08–2.66) *p* = 0.023
ECOG PS (1–2 vs. 0)	1.49 (0.81–2.76) *p* = 0.20	
Lauren (diffuse vs. intestinal)	0.78 (0.51–1.21) *p* = 0.27	
Signet ring cells (yes vs. no)	0.83 (0.49–1.42) *p* = 0.50	
Grading (G3 vs. G1–G2)	2.90 (1.60–5.27) *p* < 0.0001	1.99 (1.08–3.69) *p* = 0.028
LVI (yes vs. no)	2.11 (1.36–3.28) *p* < 0.0001	
Resection Margin (yes vs. no)	3.72 (1.83–7.57) *p* < 0.0001	2.84 (1.31–3.14) *p* = 0.008
Tumor location (medium/lower vs. upper)	0.41 (0.23–0.7637) *p* = 0.002	
Surgery (TG vs. STG)	1.73 (1.12–2.69) *p* = 0.01	1.86 (1.18–2.93) *p* = 0.008
Lymphadenectomy (D1 vs. D2)	0.61 (0.25–1.52) *p* = 0.30	
Stage (III vs. I–II)	4.32 (2.35–7.94) *p* < 0.0001	4.95 (2.50–9.81) *p* < 0.0001
T involvement (T3–T4 vs. T1–T2)	3.77 (1.73–8.20) *p* < 0.0001	
N involvement (N2–N3 vs. N0–N1)	2.40 (1.45–3.95) *p* < 0.0001	
aCT regimen (OXA vs. DG)	1.56 (0.99–2.46) *p* = 0.054	0.59 (0.35–0.99) *p* = 0.044
aRT (yes vs. no)	1.47 (0.94–2.31) *p* = 0.09	

*ECOG PS* Eastern Cooperative Oncology Group (ECOG) performance status, *TG* total gastrectomy, *STG* sub-total gastrectomy, *LVI* lymphovascular invasion, *DG* De Gramont, *OXA* XELOX/FOLFOX, *aCT* adjuvant chemotherapy, *aRT* adjuvant radiotherapy.

Regarding OS, multivariate analysis revealed that:

- Gender (HR 2.07, 95% CI: 1.28–3.34; *p* = 0.003),
- Age (HR 1.91, 95% CI: 1.21–3.02; *p* = 0.005),
- Tumor grading (HR 2.09, 95% CI: 1.11–3.94; *p* = 0.023),
- Resection margin status (HR 2.55, 95% CI: 1.25–5.24; *p* = 0.01),
- Type of surgery (HR 1.65, 95% CI: 1.06–2.58; *p* = 0.028),
- Pathological stage (HR 3.61, 95% CI: 1.90–6.87; *p* < 0.0001)

were significantly associated with survival outcomes (Table 5).

**Table 5:** Univariate and multivariate analysis for OS with selection of parameters statistically significant at the logistic regression after PSM.

	Univariate	Multivariable with selection
Gender (male vs. female)	2.14 (1.33–3.45) *p* = 0.002	2.07 (1.28–3.34) *p* = 0.003
Age (≥66 vs. <66)	2.21 (1.41–3.47) *p* < 0.0001	1.91 (1.21—3.02) *p* = 0.005
ECOG PS (1–2 vs. 0)	1.46 (0.80–2.70) *p* = 0.23	
Lauren (diffuse vs. intestinal)	0.79 (0.51–1.23) *p* = 0.30	
Signet ring cells (yes vs. no)	0.90 (0.53–1.54) *p* = 0.70	
Grading (G3 vs. G1–G2)	3.04 (1.64–5.63) *p* < 0.0001	2.09 (1.11–3.94) *p* = 0.023
LVI (yes vs. no)	2.17 (1.39–3.40) *p* < 0.0001	
Resection Margin (yes vs. no)	3.81 (1.87–7.76) *p* < 0.0001	2.55 (1.25–5.24) *p* = 0.01
Tumor location (medium/lower vs. upper)	0.44 (0.24–0.80) *p* = 0.007	
Surgery (TG vs. STG)	1.71 (1.10–2.66) *p* = 0.018	1.65 (1.06–2.58) p = 0.028
Lymphadenectomy (D1 vs. D2)	0.49 (0.18–1.34) *p* = 0.16	
Stage (III vs. I–II)	4.41 (2.36–8.25) *p* < 0.0001	3.61 (1.90–6.87) *p* < 0.0001
T involvement (T3–T4 vs. T1–T2)	3.52 (1.61–7.68) *p* < 0.0001	
N involvement (N2–N3 vs. N0–N1)	2.57 (1.54–4.29) *p* < 0.0001	
aCT (OXA vs. DG)	1.78 (1.11–2.83) *p* = 0.016	
aRT (yes vs. no)	1.58 (1.00–2.49) *p* = 0.049	

*ECOG PS* Eastern Cooperative Oncology Group (ECOG) performance status, *TG* total gastrectomy, *STG* sub-total gastrectomy, *LVI* lymphovascular invasion, *DG* De Gramont, *OXA* XELOX/FOLFOX, *aCT* adjuvant chemotherapy, *aRT* adjuvant radiotherapy.

These findings underscore the strong prognostic value of clinical, pathological, and surgical parameters independent of the adjuvant chemotherapy regimen.

## Discussion

Despite advances in GC management, a significant proportion of patients experience recurrence and mortality following surgery.

Adjuvant-CT remains a critical component to reduce recurrence risk and improve survival [4, 5].

However, the modalities of aCT differ across regions: in East Asia, it is typically administered postoperatively, whereas in Europe, perioperative chemotherapy has become the preferred strategy based on phase III trial data [6, 7].

There is broad consensus that patients with stage II–III GC should receive systemic therapy following curative surgery, whether in adjuvant or perioperative settings, depending on local practice. Eastern guidelines recommend adjuvant treatments such as S-1 monotherapy, S-1 plus docetaxel, or capecitabine plus oxaliplatin (XELOX) [26], whereas European guidelines endorse perioperative chemotherapy, particularly the FLOT regimen [27]. aCT is currently reserved for patients who undergo upfront surgery without preoperative chemotherapy. Recent studies have highlighted microsatellite instability (MSI) as a favorable prognostic marker and a potential negative predictive biomarker for aCT benefit, although prospective validation is lacking [28–30]. Our analysis found no statistically significant difference in the DFS and OS between patients treated with DG versus OXA regimens. However, after PSM a trend towards improved DFS and a significant improvement in OS was observed in the DG arm.

These results suggest that, in real-world European practice, DG and OXA regimens achieve comparable efficacy in the adjuvant setting for resected GC/GEJC. The findings align with previous reports emphasizing that patient prognosis is primarily influenced by clinical (age, gender, ECOG PS) and pathological factors (tumor grading, lymphovascular invasion, resection margin status, pathological stage), rather than the chemotherapy regimen per se.

The choice of adjuvant regimen often reflects clinical parameters, such as patient frailty, which can impact treatment compliance. Our data reinforce the importance of adequate surgery, particularly complete (R0) resection and D2 lymphadenectomy, in optimizing outcomes. Moreover, tumor location (gastro-esophageal junction versus gastric body or antrum) appears to influence prognosis, with worse outcomes observed for junctional tumors, consistent with existing literature [31, 32].

The trend toward better outcomes in the DG group warrants attention, particularly given that the XELOX regimen’s evidence base in Western populations remains limited and was largely extrapolated from the CLASSIC trial conducted in Asian cohorts. Potential explanations for these differences include variations in drug metabolism between ethnic groups, biological differences in tumor behavior, and differences in treatment adherence or tolerability.

Real-world studies like ours, although retrospective, provide valuable insights into routine clinical practice, highlighting that treatment effectiveness may differ between populations compared to controlled clinical trials. Several limitations must be acknowledged. The retrospective design, residual imbalances despite PSM, and the extended time frame of data collection (2001–2018)—during which multidisciplinary management standards evolved—all potentially impact the study’s findings. Moreover, our data suggest that DG may have been preferentially administered to older or frailer patients, which could have introduced selection bias.

Moreover, the absence of data regarding MSI and the pharmacogenetics of DPD may have influenced both the outcomes and the reported toxicity profiles.

Despite it all, the present study represents the first experience on the use of DG in a Western population and suggest that monotherapy could be considered for patients who underwent upfront surgery due to urgency, intolerance to perioperative CT or frailty. This finding is further strengthened by the absence of differences in terms of grade 3 and 4 toxicity between the OXA and the DG arms, both in the general population and in the PSM subgroups.

Overall, our results emphasize that clinical and surgical parameters—particularly age, ECOG PS, adequate lymphadenectomy, and R0 resection—remain the strongest prognostic factors in resected GC, irrespective of the adjuvant regimen. Consistent with meta-analyses, perioperative chemotherapy appears superior to postoperative aCT, particularly in Western populations [33]. In the modern era, multidisciplinary team management, patient characteristics, and emerging biomarkers such as MSI status are crucial for individualizing treatment strategies. Future prospective trials, along with the development of predictive biomarkers through immunoscore, radiomics, liquid biopsy, and molecular profiling [34–37], are needed to refine the selection of patients most likely to benefit from adjuvant therapies.

Our findings may assist clinicians in selecting appropriate adjuvant strategies for patients who are unsuitable for intensive perioperative regimens, due to comorbidities, frailty, or understaged disease at diagnosis. Furthermore, our data confirm that in elderly, frail patients, treatment with monotherapy fluoropyrimidine, commonly used in clinical practice to avoid excessive toxicity, treatment interruptions, or discontinuations, could represent a recommended practice.

## Conclusions

In this retrospective, real-world study, DG and OXA regimens demonstrated comparable efficacy as aCT for resected GC and EGJC in a European population.

After PSM, a trend toward improved DFS and a statistically significant OS benefit were observed in the DG group. These findings suggest that clinical, pathological, and surgical factors—such as patient age, ECOG performance status, resection margin status, and pathological stage—play a more significant prognostic role than the specific adjuvant chemotherapy regimen administered.

Our results highlight the need for careful patient selection and optimization of surgical outcomes to improve long-term survival in GC/EGJC. Furthermore, they emphasize the necessity of tailoring adjuvant strategies based on individual patient characteristics and emerging biological markers.

Prospective, multicenter trials specifically designed for Western populations are war-ranted to confirm these observations and to better define the optimal adjuvant approach in resected gastric cancer.

## Data Availability

The data that support the findings of this study are available from AS MD, but restrictions apply to the availability of these data, which were used under license for the current study, and so are not publicly available. Data are however available from the authors upon reasonable request and with permission of Fondazione Policlinico A. Gemelli IRCCS and University Hospital of Modena.
